# Age-Related Changes in Predictive Capacity Versus Internal Model Adaptability: Electrophysiological Evidence that Individual Differences Outweigh Effects of Age

**DOI:** 10.3389/fnagi.2015.00217

**Published:** 2015-11-30

**Authors:** Ina Bornkessel-Schlesewsky, Markus Philipp, Phillip M. Alday, Franziska Kretzschmar, Tanja Grewe, Maike Gumpert, Petra B. Schumacher, Matthias Schlesewsky

**Affiliations:** ^1^Cognitive Neuroscience Laboratory, School of Psychology, Social Work and Social Policy, University of South Australia, AdelaideSA, Australia; ^2^Institute of German Language and Literature I, University of CologneCologne, Germany; ^3^Department of English and Linguistics, Johannes Gutenberg UniversityMainz, Germany; ^4^Faculty of Health and Social Sciences, Fresenius University of Applied SciencesIdstein, Germany

**Keywords:** aging, predictive coding, language comprehension, event-related potentials, N400, P300, late positivity, individual alpha frequency

## Abstract

Hierarchical predictive coding has been identified as a possible unifying principle of brain function, and recent work in cognitive neuroscience has examined how it may be affected by age–related changes. Using language comprehension as a test case, the present study aimed to dissociate age-related changes in prediction generation versus internal model adaptation following a prediction error. Event-related brain potentials (ERPs) were measured in a group of older adults (60–81 years; *n* = 40) as they read sentences of the form “The opposite of black is white/yellow/nice.” Replicating previous work in young adults, results showed a target-related P300 for the expected antonym (“white”; an effect assumed to reflect a prediction match), and a graded N400 effect for the two incongruous conditions (i.e. a larger N400 amplitude for the incongruous continuation not related to the expected antonym, “nice,” versus the incongruous associated condition, “yellow”). These effects were followed by a late positivity, again with a larger amplitude in the incongruous non-associated versus incongruous associated condition. Analyses using linear mixed-effects models showed that the target-related P300 effect and the N400 effect for the incongruous non-associated condition were both modulated by age, thus suggesting that age-related changes affect both prediction generation and model adaptation. However, effects of age were outweighed by the interindividual variability of ERP responses, as reflected in the high proportion of variance captured by the inclusion of by-condition random slopes for participants and items. We thus argue that – at both a neurophysiological and a functional level – the notion of general differences between language processing in young and older adults may only be of limited use, and that future research should seek to better understand the causes of interindividual variability in the ERP responses of older adults and its relation to cognitive performance.

## Introduction

The cognitive and neural changes associated with healthy aging have attracted a great deal of attention within the domain of cognitive neuroscience. Reorganization of cognitive skills – and their neural bases – has been proposed as one possible mechanism for the preservation of cognitive abilities in older age, even in the face of age-related structural changes to the brain (e.g., Cabeza et al., 2002; Reuter-Lorenz and Lustig, 2005; Park and Reuter-Lorenz, 2009; Lindenberger, 2014; Shafto and Tyler, 2014). Recent work has examined the implications of this reorganization for the notion of hierarchical predictive coding, which has been proposed as a unified theory of brain function (Friston, 2005, 2010). The predictive coding framework assumes that the brain continually tests the accuracy of its current internal model of the world, by formulating predictions that can be matched against sensory input. If the prediction is borne out, the model is supported; if there is a prediction error (a mismatch between the predicted and the actual sensory input), the model must be updated. In regard to aging, it has been proposed that the predictive model is rendered less complex by a reduction in how readily model adaptations are undertaken in response to prediction errors (Moran et al., 2014). In other words, older adults are more likely to rely on predictive strategies that match the experience accrued over the lifespan, than to readily adapt their strategies to unexpected eventualities.

Language comprehension is a prime domain for testing assumptions about predictive processing: it has been proposed that prediction is the key mechanism that allows for language to be understood rapidly and efficiently (Pickering and Garrod, 2007; Dikker et al., 2009). Several behavioral studies on language comprehension in older adults appear to support Moran et al. (2014)’s conclusions about age-related changes in prediction, suggesting that older adults rely more strongly than younger adults on predictive strategies based on probabilistic cues (Rayner et al., 2006; DeDe, 2014). By contrast, the literature on the electrophysiology of language comprehension has mostly been argued to support a different conclusion, namely that older adults do not predict upcoming input as strongly as their younger counterparts. In the following, we briefly summarize the electrophysiological results in question, before suggesting a possible explanation for the apparent discrepancy with behavioral and domain-general findings.

In an early study that examined changes in the event-related potential (ERP) correlates of semantic expectations in adults across the lifespan, Kutas and Iragui (1998) presented participants aged 20–80 years with lead-in phrases that were either high or low in constraint (e.g., “the opposite of black” versus “a piece of furniture”); these were then followed by a congruent (e.g., “white,” “table”) or incongruent word (e.g., “peach,” “noose”). Incongruous words engendered an N400 effect (Kutas and Hillyard, 1980; Kutas and Federmeier, 2011), which showed a linear decrease in amplitude and a linear increase in latency with age.

Aiming to dissociate between top–down (i.e. predictive use of semantic context) and bottom–up (i.e., spreading activation of semantic features) contributions to these age-related changes in the N400, Federmeier et al. (2002) examined sentences such as those in (1) and (2).

(1) At the zoo, my sister asked if they painted the black and white stripes on the animal. I explained to her that they were natural features of a **zebra/donkey/poodle**.
(2) By the end of the day, the hiker’s feet were extremely cold and wet. It was the last time he would ever buy a cheap pair of **boots/sandals/jeans**.

The critical word (bold) in examples (1) and (2) either provided an expected continuation (e.g., “zebra”), an incongruous continuation with a word that was semantically associated with the expected continuation (e.g., “donkey”) or an incongruous and unassociated continuation (e.g., poodle). In addition, the degree of contextual constraint was either high (1) or low (2). Both young and older adults (mean age: 68 years) showed a three-way gradation of the N400 effect: incongruous unassociated > incongruous associated > congruous (for the young adults, this replicated the results in Federmeier and Kutas, 1999) and an interaction between congruity and contextual constraint. However, the source of the interaction differed between the participant groups: whereas the reduced N400 for associated versus unassociated incongruities was driven by the highly constraining contexts in the young adults, it was primarily due to the weakly constraining contexts in the elderly participants. From these results, Federmeier et al. (2002) concluded that young adults use semantic context to set up predictions about upcoming linguistic input and that, as a by-product of these predictions, semantic associates of the target item are also activated. Older adults, by contrast, use context less efficiently to set up predictions about upcoming words. This study further revealed interindividual differences, with older adults who had high verbal fluency and higher receptive vocabularies showing a similar pattern to the younger adults.

Similar conclusions were drawn from several subsequent studies. Using a modified version of Kutas and Iragui’s (1998) design that included low typicality exemplars for the low constraint (category) task [e.g., “A type of dance” – “waltz” (high typicality), “tap” (low typicality), “bait” (incongruent)], Federmeier et al. (2010) examined N400 modulations as well as modulations of a late frontal positivity. Frontal positivity effects following the N400 are observable primarily for unexpected, but possible (i.e., low cloze) continuations (for a review, see Van Petten and Luka, 2012), and have thus been linked to predictive processing. In Federmeier et al.’s (2010) experiment, young adults showed a late frontal positivity for low typicality exemplars, while older adults (60–76 years) did not show this effect as a group. However, the positivity effect was observable for older individuals with high category fluency; there was also a positive correlation between category fluency and amplitude of the frontal positivity in the older group.

These consistent electrophysiological findings show an intriguing discrepancy to the above-mentioned behavioral sentence processing (Rayner et al., 2006; DeDe, 2014) and domain-general information processing results (Moran et al., 2014), which have been interpreted as evidence for an age-related increase – rather than decrease – of predictive processing. However, since the ERP studies in question all involved the examination of (at least partial) prediction errors, there may be an alternative explanation of the data that could serve to reconcile the results across methods and domains. Specifically, rather than reflecting an age-related difference in the establishment of predictions, the frontal positivity may index age-related changes in the processing of a prediction mismatch. An explanation along these lines accords well with Moran et al.’s (2014) proposal that older adults have a lower tendency to adapt their internal predictive model on the basis of a prediction error. This proposal can also derive the observation that, in older adults, a reduced N400 for within- versus between-category violations occurs only in low-constraint (example 2) but not high-constraint (example 1) sentences. In high-constraint sentences, the predictive model is particularly strong, thus further discouraging prediction-error-based model adaptations in older adults. By contrast, when a low-constraint context does not allow for a strong prediction to be set up, model adaptations based on bottom–up input are more likely.

Distinguishing between these different accounts requires separable measures of prediction matches and prediction mismatches during sentence comprehension. While the existing electrophysiological literature on age-related changes in predictive language processing has focused primarily on consequences of failed predictions, there is, in fact, also a highly reliable electrophysiological marker of prediction *matches*: the P300. P300 effects for highly expected target words were first discussed in detail by Roehm et al. (2007b). These authors used a design similar to Kutas and Iragui’s (1998) highly constraining conditions, but formulated these as complete sentences and added a continuation that was incongruent but semantically related to the expected antonym. An example is given in (3):

(3) Das Gegenteil von schwarz ist **weiß/gelb/nett**.
  the opposite of black is **white/yellow/nice**

In young adults, sentence structures such as (3) showed an N400 effect for incongruous unassociated (nice) versus incongruous associated critical words (yellow) and a target-related P300 for congruous antonyms (white) versus incongruous associated critical words (yellow) (Roehm et al., 2007b). In this experimental setting, the P300 appears to reflect the detection of an expected target word (for similar findings in other types of sentence constructions, see Haupt et al., 2008; Molinaro and Carreiras, 2010; Vespignani et al., 2010). Roehm et al. (2007b) demonstrated the independence of the P300 from the N400 via a manipulation of the experimental task: when the same critical word pairs (black – white/yellow/nice) are presented out of context and with a task that does not render the antonym a target stimulus (lexical decision), no positivity is observable, while the N400 effect remains. In addition, the assumption of two functionally independent, but overlapping effects within the N400 time window in sentences such as (3) is supported by time–frequency analyses (Roehm et al., 2007a).

The antonym paradigm used by Roehm et al. (2007b) thus provides us with a means of separably estimating the effects of an explicit prediction match (P300 effect) and of a prediction mismatch (N400 effect). If older adults indeed use semantic context less effectively than younger adults in order to set up predictions about upcoming words, age should have a modulating effect on the P300 for the expected antonym continuations. By contrast, the N400 effect for incongruous continuations should remain unaffected. Alternatively, if model adaptation as a result of prediction errors is most strongly affected by age-related changes, the N400 effect should be most strongly modulated by age. Finally, it is possible that age-related modulations may be observable in both of these crucial aspects of predictive processing. These hypotheses were tested in the present study, which used the same experimental design and procedure as (Roehm et al., 2007b) with older adults between 60 and 81 years of age.

## Materials and Methods

### Participants

Forty older adults (20 females, 20 males; age range: 60–81 years; mean: 66.95) participated in the experiment after giving written informed consent. Two further participants were excluded due to eye-movement artifacts. All participants were healthy, right-handed, monolingual native speakers of German and had no history of reading difficulties or neurological/psychiatric disorders. Most of the participants had a tertiary education and, at the time of data acquisition, some of them were still active professionally or involved in local non-governmental or non-profit organizations.

### Materials

The stimulus materials were of the form shown in (3) and identical to those used in Roehm et al. (2007b), Experiment 1. Participants read 40 sentences per condition and 40 additional fillers involving an antonym relation, which served to counterbalance the proportion of “yes” and “no” responses in the judgment task (see below). The critical words (bold in example 3) did not differ in length or frequency across conditions (see Roehm et al., 2007b).

### Procedure

Participants were seated comfortably in front of a computer screen (19′) in a sound-attenuated room. Sentences were presented in a word-by-word manner in the center of the screen with a presentation time of 350 ms and an inter-stimulus interval of 200 ms. Participants were instructed to read all sentences attentively and to judge their plausibility by means of a button-press. As a cue for the judgment, a question mark appeared in the center of the screen 500 ms after the offset of the last word. The main experiment comprised 160 sentences in total, presented in four blocks of 40 sentences, between which participants took short breaks. The main experiment was preceded by a short practice session, in which participants were familiarized with the task.

Resting-state EEG recordings (2 min of recording with eyes open and 2 min of recording with eyes closed) were obtained from each participant at the beginning and end of the overall EEG recording session.

### EEG Recording and Preprocessing

The EEG was recorded from 27 Ag/AgCl scalp electrodes (Easycap GmbH, Herrsching-Breitbrunn, Germany) positioned according to the international 10–20 system (impedances <5 kOhm; sampling rate: 250 Hz; BrainAmp amplifier, Brain Products GmbH, Gilching, Germany). Electrodes were referenced to the right mastoid and re-referenced to linked mastoids oﬄine (ground: AFz). The electrooculogram (EOG) was recorded via bipolar pairs of electrodes placed at the outer canthus of each eye (horizontal EOG) and above and below the right eye (vertical EOG). The EEG was filtered oﬄine with 0.3–20 Hz bandpass in order to remove slow signal drifts. Raw EEGs were scanned for artifacts both automatically and manually. Automatic scanning marked all epochs as artefactual in which the EOG channels exceeded a threshold of 40 μV within a 200 ms sliding window. Manual scanning was used to mark additional artifacts, e.g., due to eye or muscle movements, signal drifts or amplifier saturation. ERPs were time-locked to the presentation onset of the critical sentence-final word. Only artifact-free trials for which the judgment task had been performed correctly entered the final data analysis. Across participants, mean trial numbers included across conditions were as follows (standard deviations in parentheses): ANT – 38.5 (2.5); REL – 38.1 (2.9); NONREL – 38.7 (2.7). The number of rejected trials did not differ across conditions: *F*(2,78) = 2.13, *p* > 0.12.^1^

### Data Analysis

For both the behavioral and the ERP amplitude data, statistical analyses were carried out using linear mixed-effects models with crossed random effects for participants and items (Baayen et al., 2008). Analyses were conducted using R (R Core Team, 2015) and the lme4 package for linear mixed-effects models (LMMs; Bates et al., 2013). Figures visualizing the fixed effects were generated using the coef2 package (Bolker and Su, 2011) and model summary tables were produced using the lmerOut package (Alday, 2015). Participants and items were modeled as random effects, whereas CONDition (expected antonym: ANT versus incongruous related: REL versus incongruous unrelated: NONREL) and AGE were modeled as fixed effects. AGE was centered via a *z*-transformation prior to inclusion in all models. The statistical analyses of the ERP data additionally included the topographical factor “region of interest” (ROI) as a fixed effect. ROIs were defined as follows: left-anterior (L-ANT): F3, F7, FC1, FC5; left-posterior (L-POST): CP1, CP5, P3, P7; right-anterior (R-ANT): F4, F8, FC2, FC6; right-posterior (R-POST): CP2, CP6, P4, P8.

In separate analyses, we included individual alpha frequency (IAF) rather than age. The IAF is the peak frequency within the EEG alpha band (8–12 Hz) during a resting-state measurement; it is known to vary among individuals (mean IAF for 30 year-old adults: 10 Hz; range 8–12) and to correlate with memory performance and intelligence (Klimesch, 1999). Recent large-scale studies of IAF variability in younger and older adults demonstrated that the association between IAF and cognitive performance remains stable across the lifespan (Grandy et al., 2013b), and that the IAF is unaffected by intensive cognitive training interventions (Grandy et al., 2013a). The IAF has thus been interpreted as a stable neurophysiological trait marker that is related to cognitive performance. We were interested in examining whether IAF is a better estimate of language processing performance than age *per se* (for findings of IAF-based differences in real-time language processing strategy, see Bornkessel et al., 2004). IAF was calculated by determining the mean peak between 7 and 12 Hz (via a Fourier transformation) in the pre- and post-experimental resting-state EEG recordings (with eyes closed) at electrodes O1 and O2. IAF was centered via a *z*-transformation prior to inclusion in all models.

For LMM modeling, we employed deviation coding for the contrasts of the factor COND, such that individual factor levels were compared to the grand mean of the dependent variable. This coding was chosen in order to allow us to separably examine the effects of a prediction match (P300 effect for ANT) and a prediction mismatch (N400 effect for NONREL), while minimizing the effects of component overlap on coefficient estimates (i.e., in these analyses, no comparisons were undertaken involving condition REL). In order to also explicitly test the hypothesis put forward by Federmeier et al. (2002, 2010) that the incongruous associated condition (REL in our case) is most strongly affected by age-related changes, we performed a second analysis assessing the effects of ANT and REL, rather than NONREL. In the following, we will refer to these two contrast codings as “match–mismatch” and “match–incongruous_associated,” respectively. The significance of fixed effects was assessed using Wald χ^2^-tests (Fox, 2016) from the car package in R (Fox and Weisberg, 2011). For the factor ROI, deviation coding was chosen to assess effects for the L-POST, R-ANT, and R-POST regions, as the left-anterior region was deemed least likely to show the effects of interest (N400, P300).

In all cases, the best-fitting LMM was determined via an iterative model fitting procedure, in which a base model (including only an intercept term for the behavioral data and an intercept term and the fixed effect ROI for the ERP data) was compared to a more complex model involving the fixed effect COND and, subsequently, to models including AGE and IAF. Note that AGE and IAF were never included in the same model, as they are known to correlate (Klimesch, 1999). Improvements of model fit were assessed using likelihood ratio tests, performed on models fit using maximum likelihood estimation (for individual models, we report fits based on restricted maximum likelihood estimation). A similar procedure was adopted to determine the most appropriate random effects structure. Models were initially fit with random intercepts for participants and items and subsequently compared to more complex models involving by-condition random slopes for participants and items. While Barr et al. (2013) recommend the general use of maximal random effects structures in order to avoid anticonservative model estimates, this may lead to model overparameterization and, hence, potential problems with model interpretability (Bates et al., 2015). Accordingly, we only adopted models with more complex random effects structures when they improved the overall fit of the model to the data.

Finally, we analyzed the peak latencies of the P300 and N400 effects in order to examine possible age-related and IAF-related latency shifts. For each participant, N400 peak latency was identified as the most negative peak between 300 and 500 ms in the difference wave obtained by subtracting condition NONREL from condition REL for the average of the posterior electrodes (P7, P3, Pz, P4, P8), at which the N400 typically shows its maximal amplitude. P300 peak latency was identified as the most positive peak between 300 and 500 ms in the difference wave obtained by subtracting condition ANT from condition REL, again for the average of the posterior electrodes [again, based on the typical distribution of P300 (P3b) effects]. We did not analyze the peak latency of the late positivity, as this effect did not show a clear peak in the grand average ERP. Possible age-related changes in peak latency values were examined via linear regression analyses.

### Ethics Statement

The experiment was performed in accordance with the Declaration of Helsinki and approved by the ethics committee of the Research Focus on Interdisciplinary Neurosciences at the Johannes Gutenberg-University Mainz.

## Results

### Individual Alpha Frequency

An IAF value was calculable for 30 of the 40 participants in this study (mean: 9.6 Hz, range: 7.7–11.1 Hz). The participants for whom IAF was not calculable did not show a clear frequency peak within the alpha range. See Supplementary Figures S12 and S13 for examples. Accordingly, all analyses involving IAF that are reported below include only the subset of 30 participants with a calculable IAF.

### Behavioral Data

Participants were very accurate in performing the judgment task. Mean accuracies per condition (with by-participant standard deviations given in parentheses) were as follows: ANT 98.62% (2.19); REL 91.99% (7.72); NONREL 99.75% (0.76). In view of the very high accuracy for all conditions in the judgment task, no inferential statistics were calculated, in order to avoid possible overinterpretations of ceiling effects.

**FIGURE 1:**
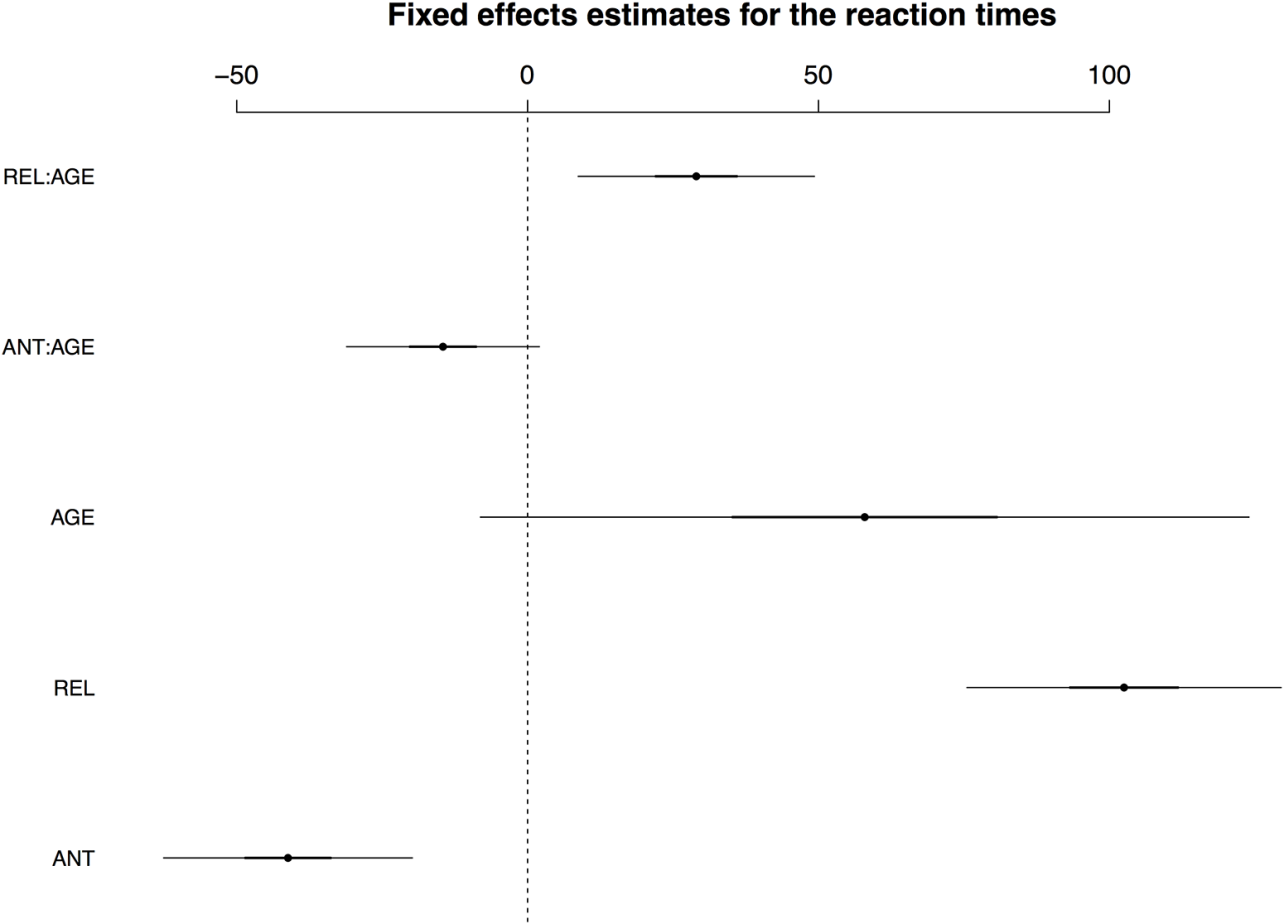
**Visualisation of fixed effects estimates for the best-fitting model for the reaction times.** Dots represent the estimated coefficient value; lines show standard deviations of coefficients (black lines) and 95% confidence intervals (CIs, 2 standard deviations; grey lines). Note that when the CI for a given effect does not cross the zero mark, this effect is considered statistically significant.

Mean reaction times (calculated for correctly answered trials only) were as follows (by-participant standard deviations are given in parentheses): ANT 642 ms (221); REL 781 ms (247); NONREL 622 ms (211). As the REL condition was the main source of variability in the reaction times, LMM analyses were only performed using the match–incongruous_associated contrasts (i.e., comparing ANT and REL to the grand mean of the reaction times). Iterative model fits revealed that a model including the fixed factors COND and AGE as well as their interaction, and per-condition random slopes for participants and items showed the best fit to the data. The fixed effects are visualized in **Figure 1**; for a full model specification and Wald χ^2^–statistics for the fixed effects in the model, see Supplementary Tables S1 and S2, respectively. The main effect of COND and the interaction of COND × AGE reached significance, with the interaction resulting from an effect of age particularly on the REL condition.

While the inclusion of IAF also led to an improved model fit, this was less substantial than that produced by age. For the parameters of the best-fitting model including IAF, see Supplementary Table S3.

### ERP Data

**FIGURE 2:**
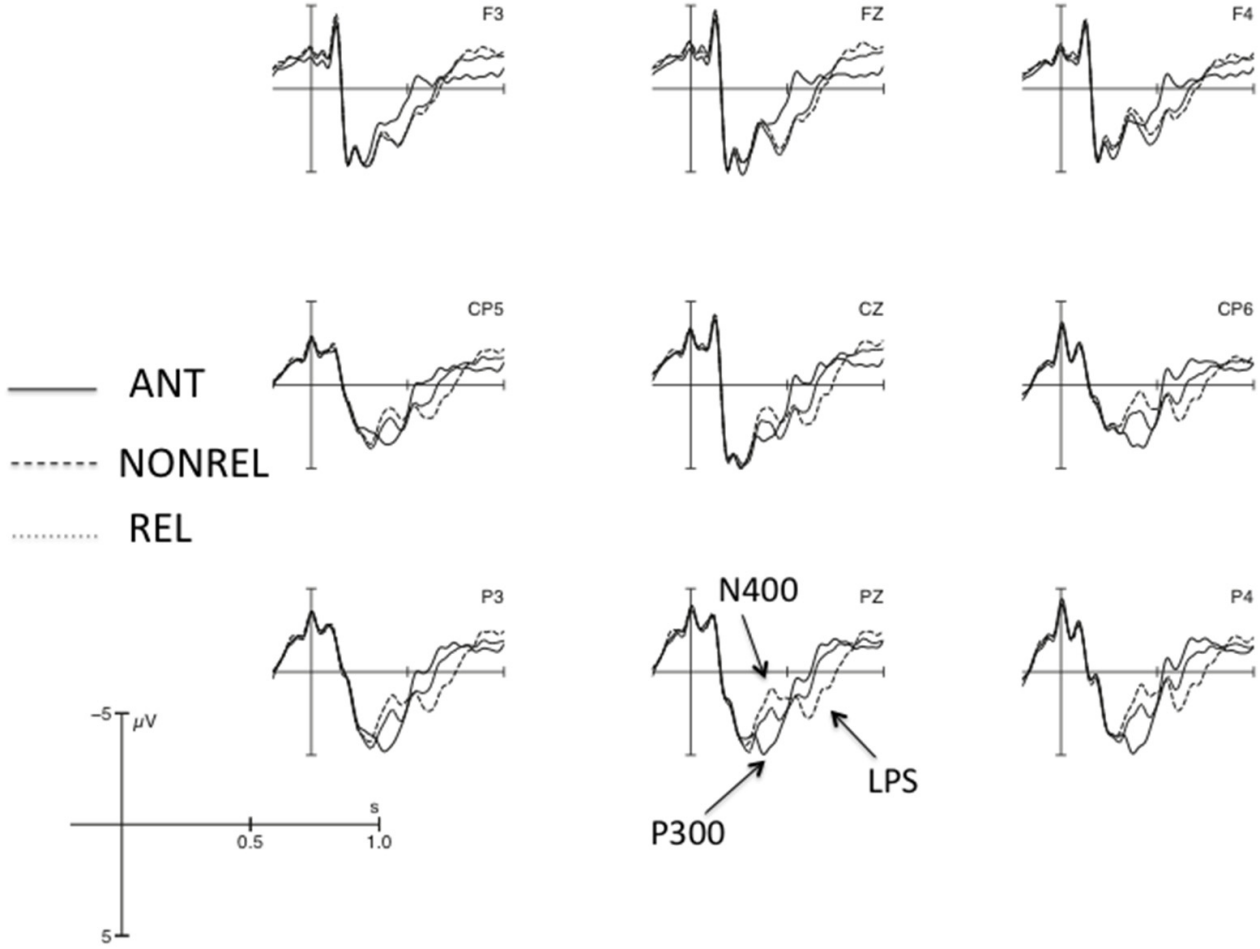
**Grand average ERPs at the position of the critical word (onset at the vertical bar) for nine selected electrodes.** Negativity is plotted upwards.

Grand average ERPs at the position of the critical word are shown in **Figure 2** for nine selected electrodes. Visual inspection of the figure suggests that the general pattern of results replicated that observed by Roehm et al. (2007b): between approximately 350 and 500 ms, there is a three-way gradation of responses, with a pronounced P300 peak for ANT and a clear N400 peak for NONREL, and REL showing an intermediary response; this is followed by a graded late positivity (LPS; NONREL > REL > ANT) between approximately 500 and 800 ms. Amplitude analyses were undertaken for the 350–500 ms (N400/P300) and 500–800 ms (LPS) time windows.

#### N400/P300 (350–500 ms)

**FIGURE 3:**
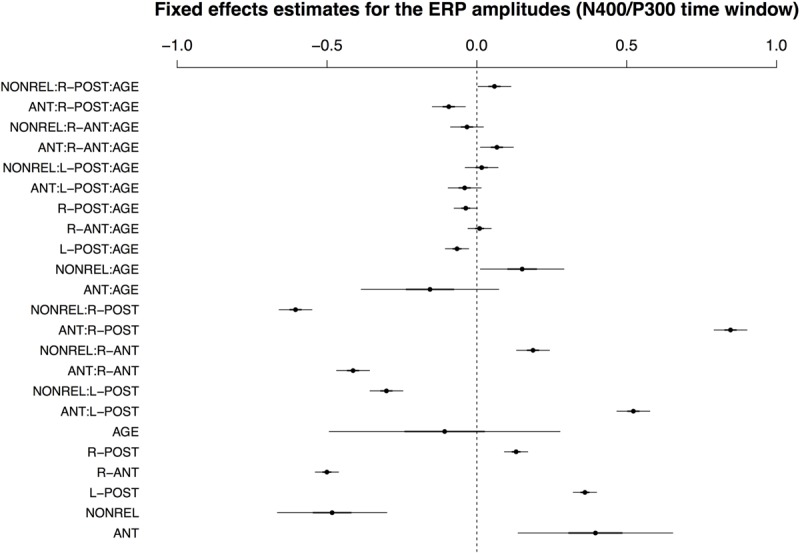
**Visualisation of fixed effects estimates for the best-fitting model of ERP amplitudes in the N400/P300 time window.** See **Figure 1** for a guide to interpreting the figure.

For the 350–500 ms time window, the best-fitting LMM included AGE and by-condition random slopes for participants and items. The fixed effects for the best-fitting model are visualized in **Figure 3**; for a full model specification and Wald χ^2^–statistics for the fixed effects in the model, see Supplementary Tables S4 and S5, respectively. As expected from our previous work, the data showed a main effect of COND and an interaction of COND × ROI. The interaction resulted from a pronounced positivity for condition ANT and a negativity for condition NONREL in posterior ROIs, but an amplitude reversal at anterior sites. Here, antonyms showed more negative-going ERP responses. For present purposes, however, interactions between COND and AGE are of particular interest. Notably, the three-way interaction COND × ROI × AGE reached significance, resulting from an interaction between COND and AGE for both ANT and REL, particularly in the right-posterior ROI.

Note that, though AGE showed a significant interaction with COND and ROI, these effects were relatively small. This resulted, in part, from the inclusion of random slopes in the model: estimates for the AGE × COND interaction in particular were considerably larger in the model without random slopes (Wald test for the AGE × COND interaction effect in the model with random slopes: χ^2^(2) = 4.71, *p* < 0.10; model without random slopes: χ^2^(2) = 115.72, *p* < 0.0001). This observation suggests that the large apparent effects of AGE in the model without random slopes are, to a considerable extent, subsumed by interindividual variability in the ERP responses. This variability appears to specifically affect the interaction of AGE and COND, as estimates for the ROI × COND interaction remain comparable in magnitude across the two models (Wald test for the ROI × COND interaction effect in the model without random slopes: χ^2^(2) = 1980.57, *p* < 0.0001; model with random slopes: χ^2^(2) = 2102.39, *p* < 0.0001).

**FIGURE 4:**
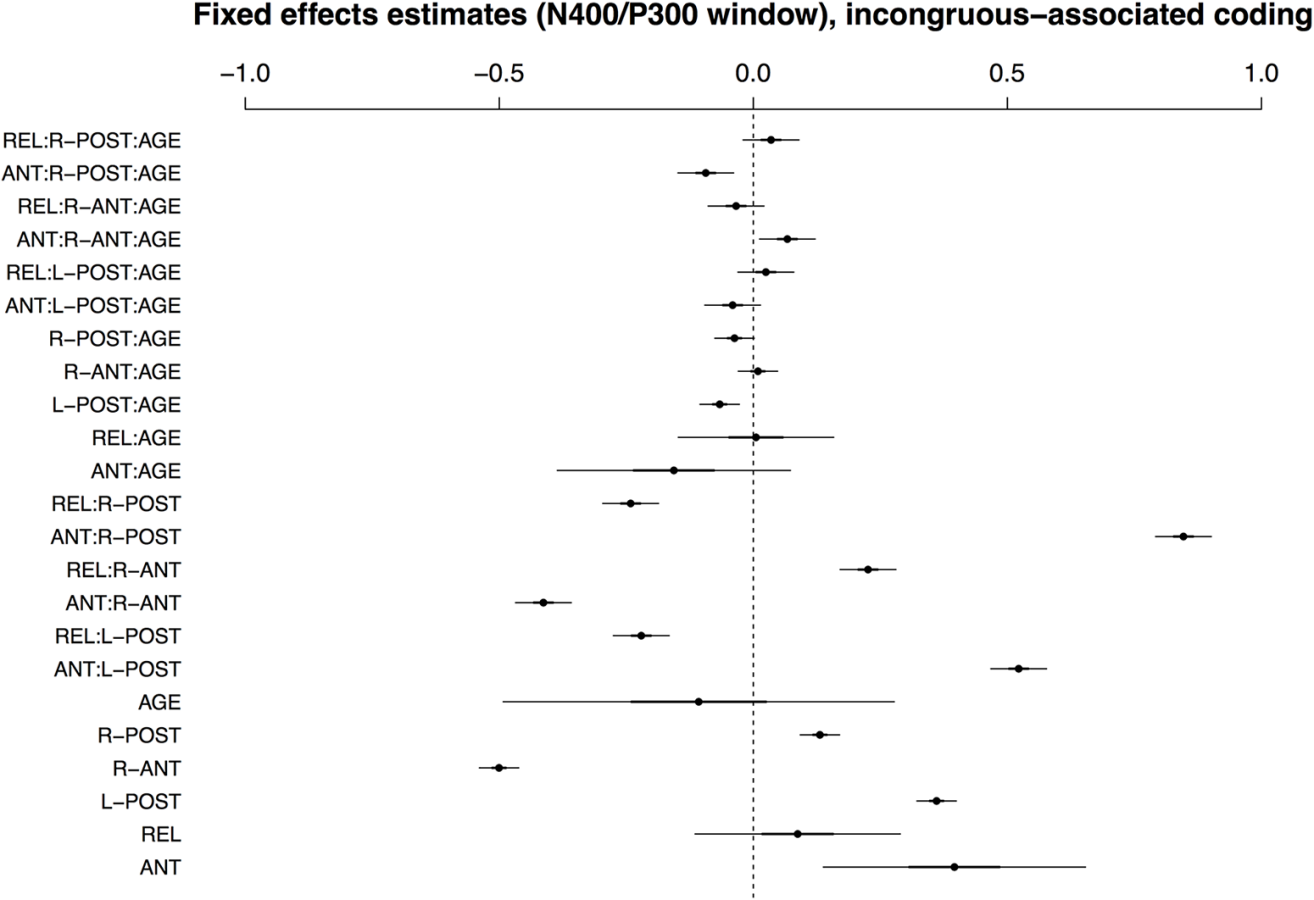
**Visualisation of fixed effects estimates for ERP amplitudes in the N400/P300 time window in the model using incongruous-associated coding.** See **Figure 1** for a guide to interpreting the figure.

**Figure 4** visualizes the fixed effects for the best-fitting model in the early time window with incongruous–associated coding (i.e., comparing effects of conditions ANT and REL to the grand mean). A full model specification is provided in Supplementary Table S6. Without consideration of AGE, the effects are similar to those in the match–mismatch encoding model described above; the interaction of COND and ROI reflects a positivity for ANT and a negativity for REL in posterior ROIs, and an amplitude reversal at anterior sites. In regard to the effects of AGE, however, the two models differ: there is no significant effect of AGE on condition REL.

For the 350–500 ms time window, the inclusion of IAF as a predictor improved model fit, but to a lesser degree than AGE. For the parameters of the best-fitting model including IAF, see Supplementary Table S7.

#### LPS (500–800 ms)

**FIGURE 5:**
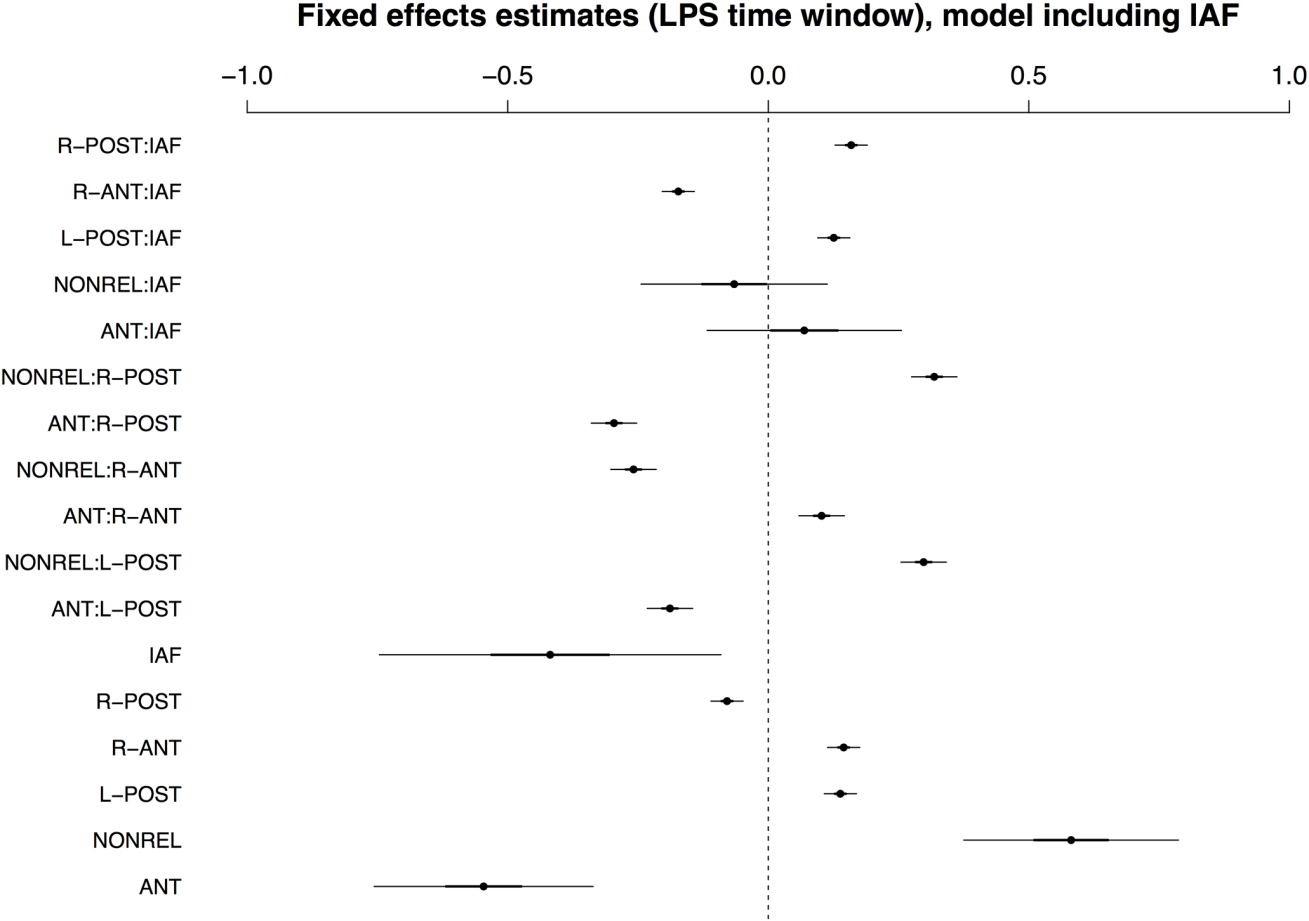
**Visualisation of fixed effects estimates for the best-fitting (IAF) model of ERP amplitudes in the LPS time window.** See **Figure 1** for a guide to interpreting the figure.

In contrast to the earlier time window, the best-fitting LMM for the late positivity window included IAF rather than COND. As with all previous models, by-condition random slopes for participants and items substantially improved model fits. The three-way interaction of COND × ROI × IAF did not reach significance and was removed from the model without a significant loss of explanatory capacity. Accordingly, the minimal adequate model for the LPS time window included IAF and by-condition random slopes, but no three-way interaction. The fixed effects in the best-fitting model are shown in **Figure 5** (see Supplementary Tables S8 and S9 for a full model summary and Wald χ^2^–statistics for the fixed effects in the model, respectively). As is apparent from the figure, the main effect of COND and the interaction COND × ROI reached significance, with the interaction due to a positivity for the NONREL condition and a relative negativity for the ANT condition in posterior ROIs. At anterior sites, there is an amplitude reversal, which appears to result from effects in the later part of the time window (i.e., a relative negativity for the ANT condition in comparison to the other two conditions; see **Figure 2**). While there was a main effect of IAF and an interaction of IAF × ROI, there was no interaction between IAF and COND.

**FIGURE 6:**
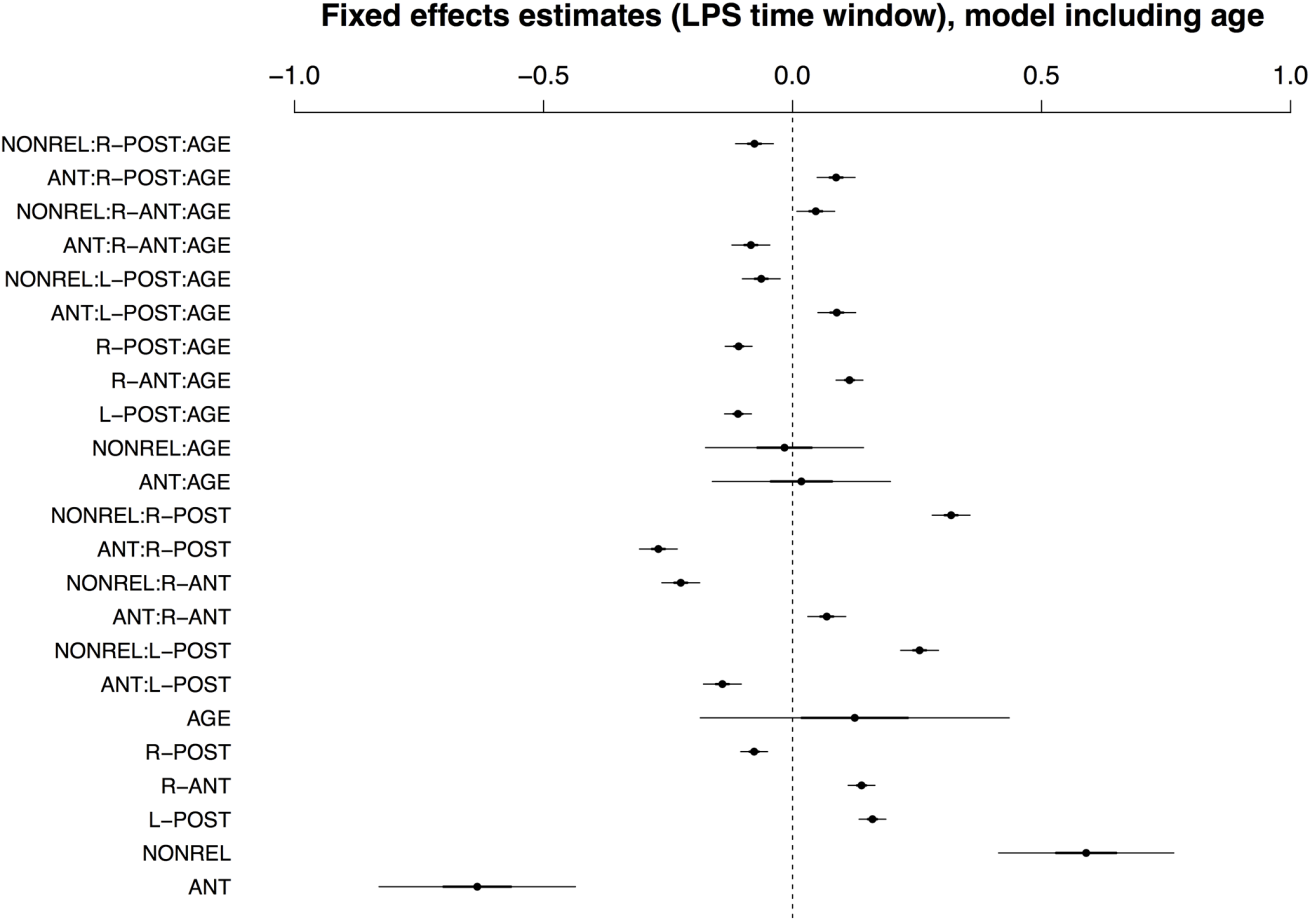
**Visualisation of fixed effects estimates for the best-fitting model of ERP amplitudes in the LPS time window including Age.** See **Figure 1** for a guide to interpreting the figure.

Though IAF outperformed AGE as a predictor in this time window, recall that the IAF dataset only covers a subset of the full sample of participants, because we were unable to reliably calculate an IAF value for 10 of the 40 participants. We thus also report the best-fitting model for the full sample, which included age and by-condition random slopes for participants and items. Fixed effects are summarized in **Figure 6**; see Supplementary Tables S10 and S11 for a full model specification and Wald χ^2^–statistics for the fixed effects in the model, respectively. In contrast to IAF, AGE did interact with COND and ROI in the late time window, with effects of AGE apparent for conditions ANT and NONREL.

#### Latency Analyses

**FIGURE 7:**
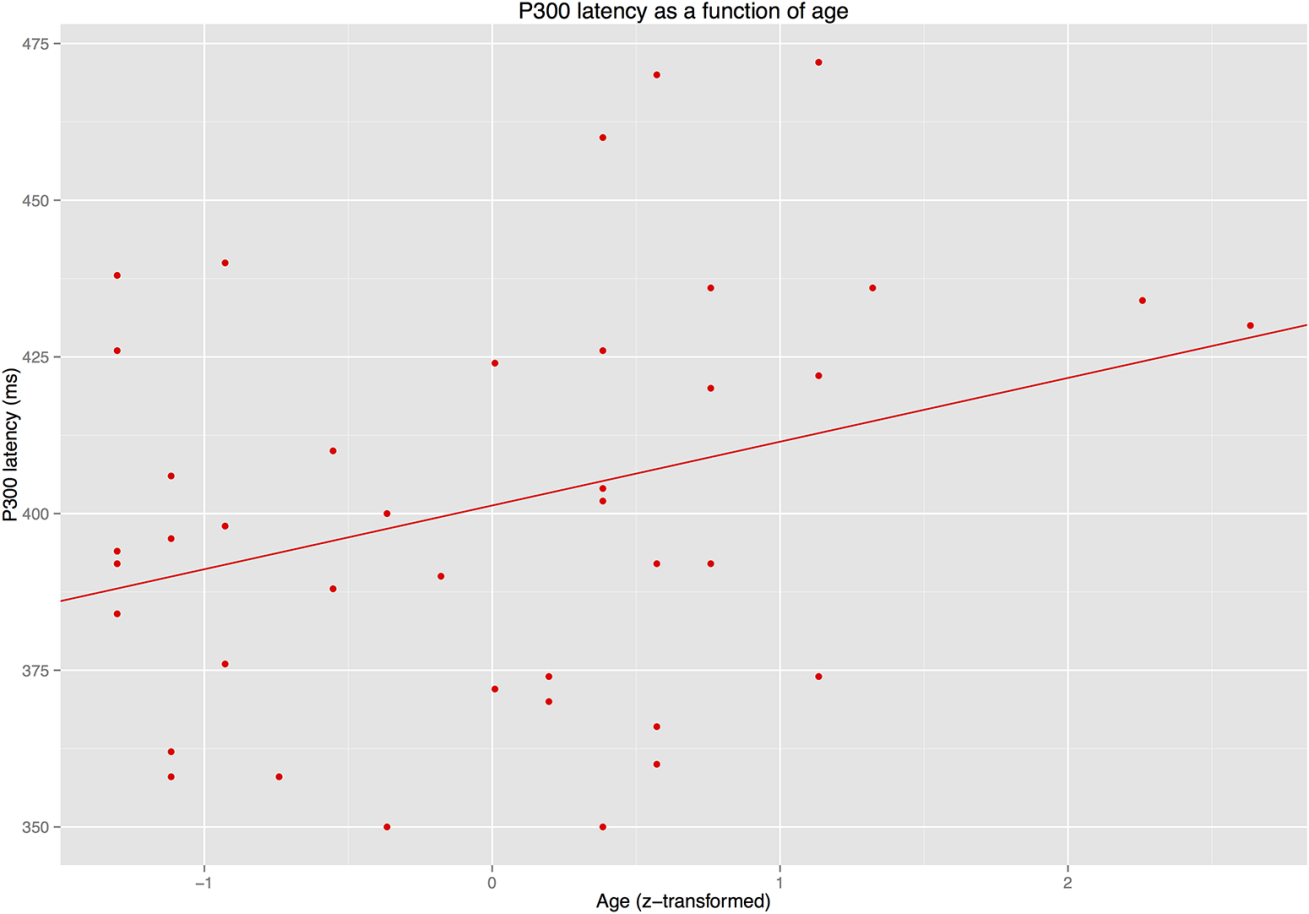
**Latency of the P300 as a function of age**.

**FIGURE 8:**
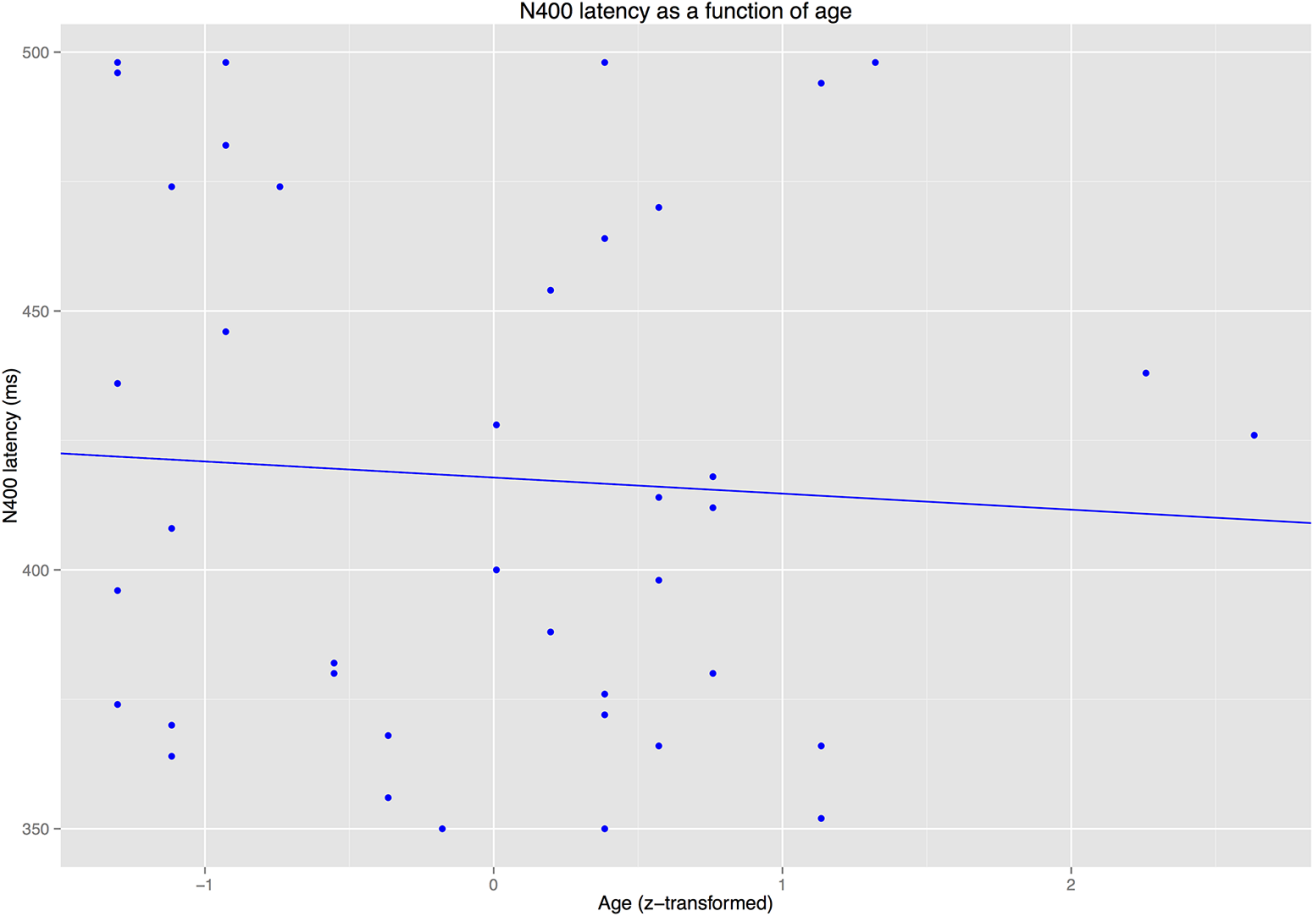
**Latency of the N400 as a function of age**.

The latency analyses for the N400/P300 time window revealed that IAF did not predict latency of the P300 or the N400 (*p*s > 0.3). By contrast, AGE predicted P300 latency (see **Figure 7**) but not N400 latency (see **Figure 8**).

## Discussion

The present study aimed to dissociate the age-related modulation of prediction matches (as reflected in a target-related P300 effect) and prediction mismatches (as reflected in an N400 effect) in real-time language comprehension. Linear mixed model (LMM) analyses of ERP results from a group of older adults (60–81 years) revealed that both effects were modulated by age. Crucially, however, only the N400 effect for prediction mismatches that were not associated with the expected continuation (condition NONREL) was modulated by age, while that for semantically associated prediction mismatches (REL) was not. Age-related latency shifts were only observed for the target-related P300, but not for the N400. In a later time window, late positivity effects for both prediction mismatch conditions were modulated by age. However, the best-fitting model for the late time window included IAF rather than age, though IAF did not interact with condition. In the following, we first discuss the effects in the N400/P300 time window, as these were most central to the hypotheses examined in this study, before turning to the late positivity and more general implications of these results.

### Age-related Changes in Predictive Processing and the Effects of Interindividual Variability

The results of the current study suggest that age-related changes in predictive processing affect both the prediction itself (as reflected in a reduced P300 effect for prediction matches with age) and the model adaptation resulting from a prediction mismatch (as reflected in a reduced N400 effect for prediction mismatches with age).

However, the magnitude of these age-related effects was far outweighed by the individual variability among participants, as shown by the substantial decrease in effect estimates for the AGE × COND interaction once random slopes were included in the model. The fact that no comparable decrease was observable for the ROI × COND interaction suggests that the interindividual variability particularly affected the estimability of age-related influences on ERPs. This observation has potential implications for the interpretation of existing findings on aging and the electrophysiology of language processing. Most previous studies in this domain have compared groups of younger and older adults using analyses of variance (ANOVAs). It appears possible that the age-related decreases in ERP amplitudes observed in this way (or, more drastically, the apparent absence of an ERP effect of interest in older adults; e.g., Delong et al., 2012) result, at least in part, from a larger degree of interindividual variability in the older group.

Our results indicate that the assumption of general differences between the electrophysiological responses of younger and older adults may be of limited use for understanding age-related changes in cognitive processing. Rather, we suggest that it may be more fruitful to study how the high individual variation in the ERP patterns of older adults relates to individual differences in processing strategies and the success of these strategies for effective cognitive processing. In this regard, our results support and further extend the finding by Federmeier et al. (2002, 2010) that a subset of older adults – namely those with higher verbal fluency – showed an ERP response pattern resembling that of young adults, while the ERPs of the older adults as a group differed from those of the young adults. It is a limitation of the present study that, since we did not collect neuropsychological data from our participants, we are unable to determine whether the high degree of inter-individual variability could potentially be explained – at least in part – by measures such as verbal fluency. This is a clear objective for future studies in this area.

In another respect, however, our results are not in agreement with Federmeier and colleagues’ findings. Federmeier et al. (2002, 2010) drew their conclusions regarding age-related changes in predictive processing on the basis of the finding that ERP responses to incongruous-associated continuations – i.e., words that were unexpected, but semantically related to the expected continuation – were particularly susceptible to changes in older adults. The results of the present study suggest that apparent changes in incongruous-associated conditions (REL in the present study) may, in fact, result from age-related changes in two overlapping ERP components for the conditions to which the incongruous-associated condition must be compared: the target-related P300 for a predicted continuation (ANT) and the N400 for an unassociated prediction mismatch (NONREL). Using a statistical analysis technique that was designed to minimize the influence of effect co-dependencies between individual conditions, we observed age-related effects in conditions ANT and NONREL, but not REL. The necessity of considering both the P300 and the N400 in experimental designs of this type is further emphasized by the observation of an age-related latency shift in the P300, but not the N400 in the present study, a finding that further corroborates our previous results on the separability of the two components (Roehm et al., 2007a,b).

A potential caveat with regard to our conclusion that age-related changes in language-related ERPs are outweighed by interindividual variability is that the current study only examined a sample of older adults (60–81 years), rather than testing participants across a broader age range. However, previous studies of extended lifespan samples (20–80 years) have found evidence for linear changes in N400 and P300 amplitude and latency across the lifespan (Picton et al., 1984; Kutas and Iragui, 1998), thus suggesting that the 20-year age range examined here should have been sufficient to show such changes if present. The possibility of non-linear change patterns has not been explored in the ERP literature on language processing and aging to date, in spite of evidence for non-linear age-related changes in language production (e.g., Kemper et al., 2001). Future studies in the ERP domain should consider this possibility.

### The Late Positivity and Effects of Individual Alpha Frequency and Age

In terms of the general pattern observed, the late positivity effects in the present study replicated those observed in our earlier experiment with young adults (Roehm et al., 2007b). More interesting, however, are the effects of age and IAF observed in this time window.

In contrast to the earlier time window, IAF outperformed age in improving model fit for the late positivity. Intriguingly, the improvement of model fit was not accompanied by an interaction between IAF and COND. Rather, the substantial IAF × ROI interaction suggests that inclusion of IAF was able to account for topographical variability in our participants’ overall ERP responses, irrespective of our experimental manipulation. While we can only speculate regarding the functional significance of this observation, one possibility is that it is related to the role of alpha oscillations in long-range information transfer in the brain (e.g., Canolty and Knight, 2010) and in the timing of information processing via changes in inhibitory state (Klimesch et al., 2007). In individuals with a higher IAF, the precision of this timing is increased, thus leading to a more efficient transfer of information between distributed brain regions (Grandy et al., 2013b). Consequently, IAF could account for individual differences in topographical variability of ERP responses.

By contrast, the best-fitting model including age did show interactions of AGE, ROI, and COND. As in the N400/P300 time window, age-related differences were observed for both ANT and NONREL, thus further emphasizing the need to examine mechanisms of both prediction matches and prediction mismatches when considering age-related changes in predictive processing. The dissociation between age, which led to a modulation of the ERP effects in our critical conditions, and IAF, which did not interact with our experimental manipulation but led to a greater improvement in overall model fit than age, is intriguing and indicates that, though age and IAF are correlated, they reflect different things. While age may be more strongly correlated with individual changes in processing strategies, IAF has been argued to be a neurophysiological trait marker that is related to aspects of cognitive performance (Grandy et al., 2013a,b). Its predictive capacity is retained across the lifespan (or at least approximately up to the age of 80) (Grandy et al., 2013b). This functional dissociation may be related to the different levels of specificity with which age and IAF interacted with the other factors in our experiment.

## Conclusion

With the present study, we aimed to shed further light on which aspects of predictive processing are particularly affected by age-related changes: those related to setting up the prediction or those related to adapting the internal model in response to a prediction mismatch. While our neurophysiological findings provide some initial indications that both types of processes are modulated by age, we consider more significant the result that age-related modulations of language-related ERP responses are considerably smaller than the variability observed across individual participants. This observation suggests that – at both a neurophysiological and a functional level – interindividual differences between older adults should be taken into account more strongly in the interpretation of seemingly general age-related differences in language processing. Future research should seek to better understand the causes underlying the high degree of variability in the event-related electrophysiological responses of older adults and how it relates to cognitive performance.

## Author Contributions

IB-S, MP, TG, PS, and MS designed the research. MP and MG acquired the data. IB-S, MP, PA, FK, and MS analyzed the data. All authors contributed to data interpretation and to the writing of the paper.

## Conflict of Interest Statement

The authors declare that the research was conducted in the absence of any commercial or financial relationships that could be construed as a potential conflict of interest.

## Footnotes

1 For the individual alpha frequency (IAF) analysis (see Data Analysis), trial numbers were as follows: ANT – 38.3 (2.7); REL – 37.7 (3.2); NONREL – 38.4 (3.0). Again, there were no significant differences across conditions: *F*(2,58) = 2.50, *p* > 0.09.
