# Supplementary material for: Age-Related Changes in Predictive Capacity Versus Internal Model Adaptability: Electrophysiological Evidence that Individual Differences Outweigh Effects of Age
Source: Front Aging Neurosci. 2015 Nov 30;7:217. doi: 10.3389/fnagi.2015.00217 (PMC4663277; doi:10.3389/fnagi.2015.00217)

Table S1: Model parameters for the best-fitting model for the reaction times

Linear mixed model fit by REML

REML criterion at convergence: 65776

Scaled residuals:

Min	1Q	Median	3Q	Max
-3.39	-0.51	-0.11	0.31	7.28

Random effects:

Groups	Name	Variance	Std.Dev	Corr
itm	(Intercept)	2172.03	46.61	
itm	ANT	3760.58	61.32	−0.29
itm	REL	6561.05	81.00	−0.82
subj	(Intercept)	44897.18	211.89	
subj	ANT	1111.50	33.34	−0.07
subj	REL	2463.75	49.64	−0.89
Residual		100873.97	317.61	

Number of obs: 4560, groups: itm, 80; subj, 40.

Fixed effects:

	Estimate	Std. Error	t value
(Intercept)	6.8e+02	34	20
ANT	−41	11	−3.8
REL	1e+02	14	7.4
age	58	34	1.7
ANT:age	−15	8.5	−1.7
REL:age	29	10	2.8

Table S2: Wald tests for the reaction time model in Table S1

	χ^2	Df	$Pr(> \chi^2)$	
cond	62.05	2	< 0.001	***
age	1.35	1	0.246	
cond:age	7.90	2	0.0193	*

Table S3: Model parameters for the best-fitting model for the reaction times including IAF

Linear mixed model fit by REML

REML criterion at convergence: 49069

Scaled residuals:

Min	1Q	Median	3Q	Max
-3.25	-0.51	-0.12	0.31	7.69

Random effects:

Groups	Name	Variance	Std.Dev	Corr
itm	(Intercept)	1898.04	43.57	
itm	ANT	2853.16	53.41	−0.26
itm	NONREL	6897.86	83.05	−0.88
subj	(Intercept)	31840.35	178.44	
subj	ANT	788.89	28.09	−0.26
subj	NONREL	1920.80	43.83	−0.80
Residual		94197.88	306.92	

Number of obs: 3419, groups: itm, 80; subj, 30.

Fixed effects:

	Estimate	Std. Error	t value
(Intercept)	6.8e+02	33	20
ANT	−42	11	−3.9
NONREL	1e+02	14	7
iaf	−83	33	−2.5
ANT:iaf	18	9.1	2
NONREL:iaf	−27	11	−2.4

Table S4: Model parameters for the best-fitting model for ERP amplitudes in the N400/P300 time window (350-500 ms)

Linear mixed model fit by REML

REML criterion at convergence: 377837

Scaled residuals:

Min	1Q	Median	3Q	Max
-12.33	-0.61	0	0.62	24.62

Random effects:

Groups	Name	Variance	Std.Dev	Corr
itm	(Intercept)	0.41	0.64	
itm	ANT	0.29	0.54	−0.37
itm	NONREL	0.29	0.54	−0.37
subj	(Intercept)	1.54	1.24	
subj	ANT	0.54	0.73	−0.00
subj	NONREL	0.19	0.44	−0.77
Residual		9.66	3.11	

Number of obs: 73744, groups: itm, 80; subj, 40.

Fixed effects:

	Estimate	Std. Error	t value
(Intercept)	2.1	0.21	10
ANT	0.4	0.13	3
NONREL	−0.48	0.093	−5.2
L-POST	0.36	0.02	18
R-ANT	−0.5	0.02	−25
R-POST	0.13	0.02	6.6
age	−0.11	0.2	−0.55
ANT:L-POST	0.52	0.028	19
NONREL:L-POST	−0.3	0.028	−11
ANT:R-ANT	−0.41	0.028	−15
NONREL:R-ANT	0.19	0.028	6.7
ANT:R-POST	0.85	0.028	30
NONREL:R-POST	−0.61	0.028	−22
ANT:age	−0.16	0.12	−1.3
NONREL:age	0.15	0.071	2.1
L-POST:age	−0.066	0.02	−3.3
R-ANT:age	0.0091	0.02	0.46
R-POST:age	−0.037	0.02	−1.9
ANT:L-POST:age	−0.041	0.028	−1.5
NONREL:L-POST:age	0.016	0.028	0.57
ANT:R-ANT:age	0.067	0.028	2.4
NONREL:R-ANT:age	−0.033	0.028	−1.2
ANT:R-POST:age	−0.094	0.028	−3.3
NONREL:R-POST:age	0.059	0.028	2.1

Table S5: Wald tests for the ERP amplitude model (350-500 ms) in Table S4

	χ^2	Df	$\Pr(> \chi^2)$	
cond	27.21	2	< 0.001	***
roi	760.46	3	< 0.001	***
age	0.36	1	0.547	
cond:roi	2102.39	6	0	***
cond:age	4.71	2	0.0949	.
roi:age	28.08	3	< 0.001	***
cond:roi:age	18.88	6	0.00438	**

Table S6: Model parameters for the best-fitting model (incongruous-associated coding) for ERP amplitudes in the N400/P300 time window (350-500 ms)

Linear mixed model fit by REML

REML criterion at convergence: 377837

Scaled residuals:

Min	1Q	Median	3Q	Max
-12.33	-0.61	0	0.62	24.62

Random effects:

Groups	Name	Variance	Std.Dev	Corr
itm	(Intercept)	0.41	0.64	
itm	ANT	0.29	0.54	−0.37
itm	REL	0.36	0.60	−0.56
subj	(Intercept)	1.54	1.24	
subj	ANT	0.54	0.73	−0.00
subj	REL	0.23	0.48	−0.82
Residual		9.66	3.11	

Number of obs: 73744, groups: itm, 80; subj, 40.

Fixed effects:

	Estimate	Std. Error	t value
(Intercept)	2.1	0.21	10
ANT	0.4	0.13	3
REL	0.087	0.1	0.85
L-POST	0.36	0.02	18
R-ANT	−0.5	0.02	−25
R-POST	0.13	0.02	6.6
age	−0.11	0.2	−0.55
ANT:L-POST	0.52	0.028	19
REL:L-POST	−0.22	0.028	−7.8
ANT:R-ANT	−0.41	0.028	−15
REL:R-ANT	0.23	0.028	8
ANT:R-POST	0.85	0.028	30
REL:R-POST	−0.24	0.028	−8.6
ANT:age	−0.16	0.12	−1.3
REL:age	0.0052	0.078	0.067
L-POST:age	−0.066	0.02	−3.3
R-ANT:age	0.0091	0.02	0.46
R-POST:age	−0.037	0.02	−1.9
ANT:L-POST:age	−0.041	0.028	−1.5
REL:L-POST:age	0.025	0.028	0.88
ANT:R-ANT:age	0.067	0.028	2.4
REL:R-ANT:age	−0.034	0.028	−1.2
ANT:R-POST:age	−0.094	0.028	−3.3
REL:R-POST:age	0.035	0.028	1.2

Table S7: Model parameters for the best-fitting model for ERP amplitudes in the N400/P300 time window (350-500 ms) including IAF

Linear mixed model fit by REML

REML criterion at convergence: 282250

Scaled residuals:

Min	1Q	Median	3Q	Max
-12.05	-0.61	0.01	0.61	24.5

Random effects:

Groups	Name	Variance	Std.Dev	Corr
itm	(Intercept)	0.40	0.63	
itm	ANT	0.39	0.63	−0.33
itm	NONREL	0.41	0.64	−0.46
subj	(Intercept)	1.79	1.34	
subj	ANT	0.37	0.61	−0.11
subj	NONREL	0.12	0.34	−0.81
Residual		9.77	3.13	

Number of obs: 54944, groups: itm, 80; subj, 30.

Fixed effects:

	Estimate	Std. Error	t value
(Intercept)	2.2	0.25	8.4
ANT	0.28	0.13	2.1
NONREL	−0.45	0.097	−4.7
L-POST	0.4	0.023	17
R-ANT	−0.53	0.023	−23
R-POST	0.28	0.023	12
iaf	0.22	0.24	0.91
ANT:L-POST	0.48	0.033	15
NONREL:L-POST	−0.26	0.033	−8
ANT:R-ANT	−0.37	0.033	−11
NONREL:R-ANT	0.14	0.033	4.2
ANT:R-POST	0.83	0.033	25
NONREL:R-POST	−0.58	0.033	−18
ANT:iaf	−0.11	0.11	−1
NONREL:iaf	0.029	0.064	0.46
L-POST:iaf	−0.037	0.023	−1.6
R-ANT:iaf	0.17	0.023	7.3
R-POST:iaf	−0.12	0.023	−5.1
ANT:L-POST:iaf	−0.068	0.032	−2.1
NONREL:L-POST:iaf	0.061	0.033	1.9
ANT:R-ANT:iaf	−0.0026	0.032	−0.081
NONREL:R-ANT:iaf	−0.022	0.033	−0.67
ANT:R-POST:iaf	0.091	0.032	2.8
NONREL:R-POST:iaf	−0.028	0.033	−0.84

Table S8: Model parameters for the best-fitting model for ERP amplitudes in the LPS time window (500-800 ms)

Linear mixed model fit by REML

REML criterion at convergence: 239976

Scaled residuals:

Min	1Q	Median	3Q	Max
-22.64	-0.62	-0.02	0.6	15.13

Random effects:

Groups	Name	Variance	Std.Dev	Corr
itm	(Intercept)	0.31	0.56	
itm	ANT	0.15	0.39	0.06
itm	NONREL	0.18	0.43	−0.49
subj	(Intercept)	0.88	0.94	
subj	ANT	0.28	0.53	0.15
subj	NONREL	0.26	0.51	−0.72
Residual		4.52	2.13	

Number of obs: 54944, groups: itm, 80; subj, 30.

Fixed effects:

	Estimate	Std. Error	t value
(Intercept)	−0.12	0.18	−0.68
ANT	−0.55	0.11	−5.1
NONREL	0.58	0.1	5.5
L-POST	0.14	0.016	8.8
R-ANT	0.14	0.016	9.2
R-POST	−0.079	0.016	−5
iaf	−0.42	0.17	−2.5
ANT:L-POST	−0.19	0.022	−8.5
NONREL:L-POST	0.3	0.022	13
ANT:R-ANT	0.1	0.022	4.6
NONREL:R-ANT	−0.26	0.022	−12
ANT:R-POST	−0.3	0.022	−13
NONREL:R-POST	0.32	0.022	14
ANT:iaf	0.069	0.095	0.73
NONREL:iaf	−0.066	0.091	−0.72
L-POST:iaf	0.13	0.016	8
R-ANT:iaf	−0.17	0.016	−11
R-POST:iaf	0.16	0.016	10

Table S9: Wald tests for the ERP amplitude model (500-800 ms) in Table S8

	χ^2	Df	$\Pr(>\chi^2)$	
cond	34.10	2	< 0.001	***
roi	267.09	3	< 0.001	***
iaf	6.94	1	0.00845	**
cond:roi	715.80	6	< 0.001	***
cond:iaf	0.61	2	0.737	
roi:iaf	253.86	3	< 0.001	***

Table S10: Model parameters for the best-fitting model for ERP amplitudes in the LPS time window (500-800 ms) including Age

Linear mixed model fit by REML

REML criterion at convergence: 323276

Scaled residuals:

	Min	1Q	Median	3Q	Max
	-22.3	-0.62	-0.02	0.61	14.92

Random effects:

Groups	Name	Variance	Std.Dev	Corr
itm	(Intercept)	0.30	0.55	
itm	ANT	0.14	0.38	0.17
itm	NONONREL	0.12	0.35	−0.56
subj	(Intercept)	1.00	1.00	
subj	ANT	0.33	0.57	−0.04
subj	NONONREL	0.26	0.51	−0.74
Residual		4.60	2.15	

Number of obs: 73744, groups: itm, 80; subj, 40.

Fixed effects:

	Estimate	Std. Error	t value
(Intercept)	0.048	0.17	0.28
ANT	−0.63	0.1	−6.3
NONONREL	0.59	0.09	6.6
L-POST	0.16	0.014	12
R-ANT	0.14	0.014	10
R-POST	−0.077	0.014	−5.6
age	0.12	0.16	0.79
ANT:L-POST	−0.14	0.019	−7.3
NONONREL:L-POST	0.26	0.019	13
ANT:R-ANT	0.069	0.019	3.5
NONONREL:R-ANT	−0.22	0.019	−12
ANT:R-POST	−0.27	0.019	−14
NONONREL:R-POST	0.32	0.019	16
ANT:age	0.018	0.091	0.19
NONONREL:age	−0.016	0.081	−0.2
L-POST:age	−0.11	0.014	−8
R-ANT:age	0.11	0.014	8.4
R-POST:age	−0.11	0.014	−7.9
ANT:L-POST:age	0.089	0.019	4.6
NONONREL:L-POST:age	−0.063	0.019	−3.3
ANT:R-ANT:age	−0.084	0.019	−4.3
NONONREL:R-ANT:age	0.047	0.019	2.4
ANT:R-POST:age	0.087	0.019	4.5
NONONREL:R-POST:age	−0.077	0.019	−4

Table S11: Wald tests for the ERP amplitude model (500-800 ms) in Table S10

	χ^2	Df	$Pr(> \chi^2)$	
cond	48.72	2	< 0.001	***
roi	401.33	3	< 0.001	***
age	0.64	1	0.425	
cond:roi	792.65	6	< 0.001	***
cond:age	0.04	2	0.978	
roi:age	190.45	3	< 0.001	***
cond:roi:age	72.12	6	< 0.001	***

Figure S12

Example of a participant with a calculable IAF (i.e. a clear frequency peak within the 7–12 Hz range). The four panels show the frequency power spectra for the resting-state EEG sessions with eyes closed: electrode O1, before the experiment (panel A); electrode O2, before the experiment (panel B); electrode O1, after the experiment (panel C); electrode O2, after the experiment (panel D). IAF was calculated as the mean of the four peaks (if present) from these four recordings.

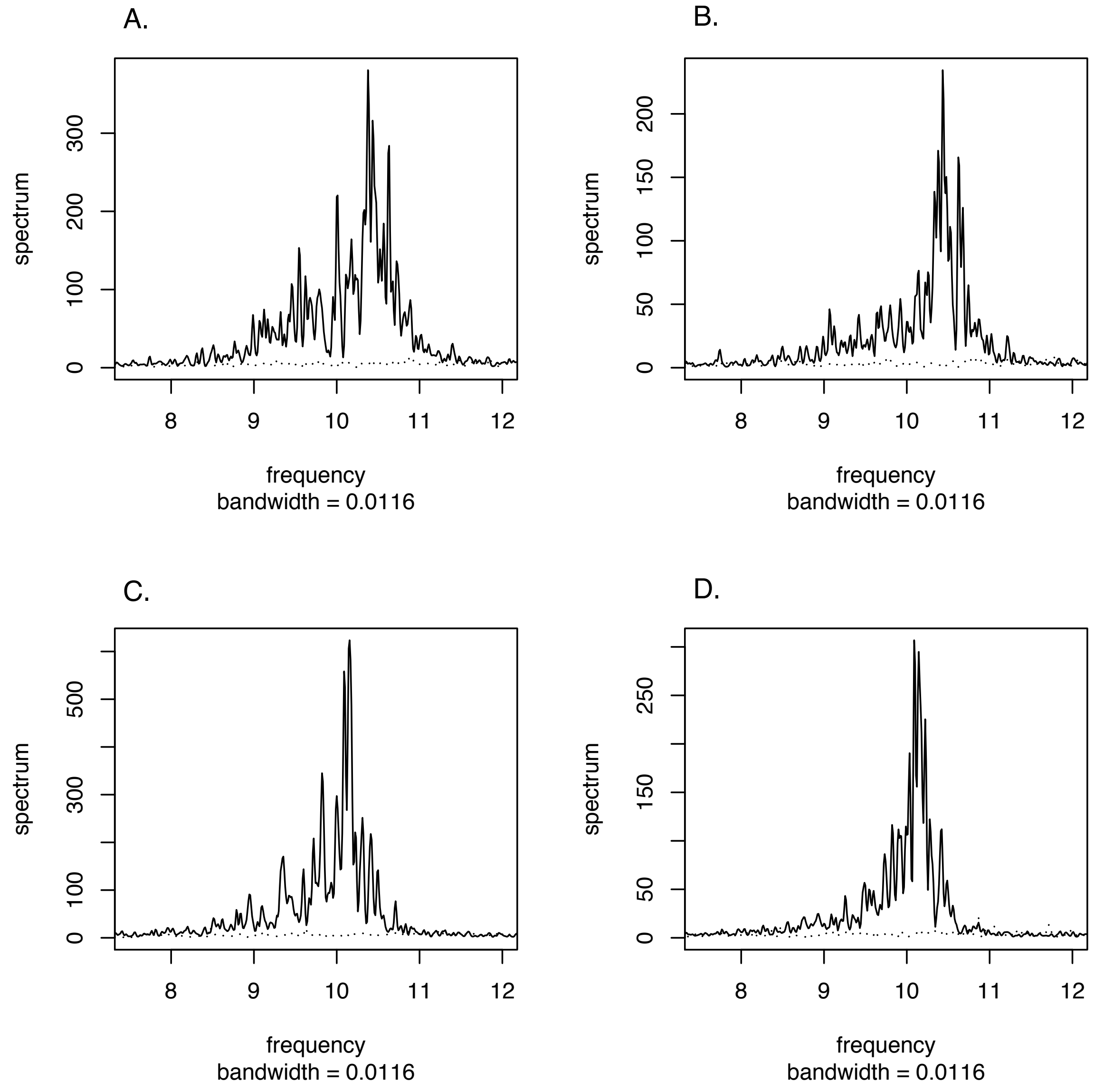

Figure S13

Example of a participant without a calculable IAF (i.e. a clear frequency peak within the 7–12 Hz range). The four panels show the frequency power spectra for the resting-state EEG sessions with eyes closed: electrode O1, before the experiment (panel A); electrode O2, before the experiment (panel B); electrode O1, after the experiment (panel C); electrode O2, after the experiment (panel D). IAF was calculated as the mean of the four peaks (if present) from these four recordings.

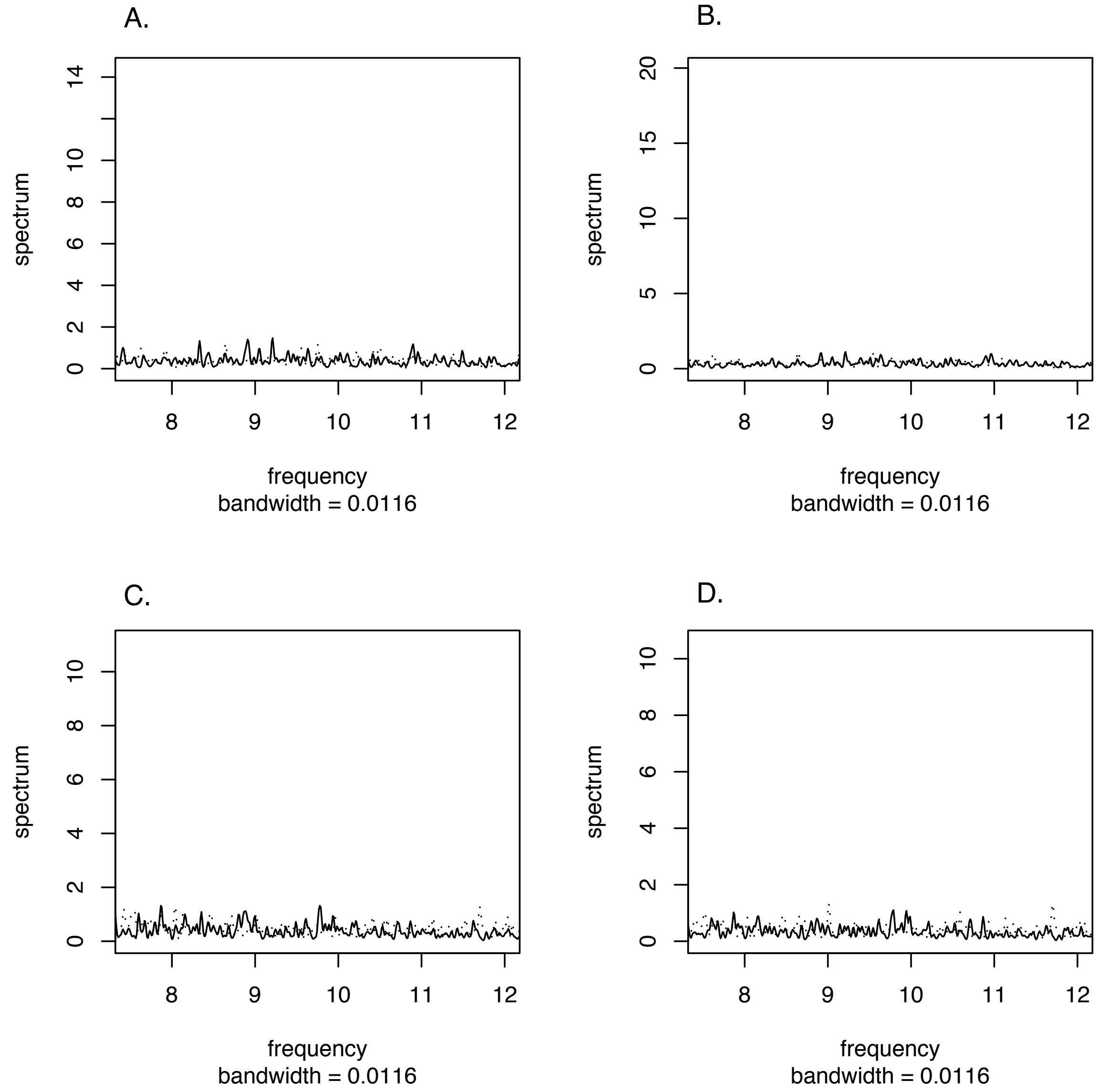

Supplement: Supplementary file 1 [file Data_Sheet_1.PDF]
